# Improving the heterotrophic media of three *Chlorella vulgaris* mutants toward optimal color, biomass and protein productivity

**DOI:** 10.1038/s41598-025-05913-1

**Published:** 2025-07-02

**Authors:** Mafalda Trovão, Miguel Cunha, Gonçalo Espírito Santo, Humberto Pedroso, Ana Reis, Ana Barros, Nádia Correia, Lisa Schüler, Monya Costa, Sara Ferreira, Helena Cardoso, Márcia Ventura, João Varela, Joana Silva, Filomena Freitas, Hugo Pereira

**Affiliations:** 1 R&D Department, Allmicroalgae Natural Products S.A, Pataias, 2445-413 Portugal; 2 GreenCoLab - Associação Oceano Verde, University of Algarve, Faro, 8005-139 Portugal; 3 Associate Laboratory i4HB - Institute for Health and Bioeconomy, School of Science and Technology, NOVA University Lisbon, Campus de Caparica, Caparica, 2829-516 Portugal; 4 UCIBIO – Applied Molecular Biosciences Unit, Department of Chemistry, School of Science and Technology, NOVA University Lisbon, Campus de Caparica, Caparica, 2829-516 Portugal; 5 Department of Chemistry, NOVA School of Science and Technology, LAQV-REQUIMTE, NOVA University Lisbon, Campus de Caparica, Ed. Departamental, Piso 5, Caparica, 2829-516 Portugal; 6 CCMAR - Centre of Marine Sciences, University of Algarve, Faro, 8005-139 Portugal

**Keywords:** Biomass color, Biomass and protein productivity, Culture medium optimization, Microalgae, Random mutagenesis, Surface response methodology, Biochemistry, Biological techniques, Microbiology, Applied microbiology, Industrial microbiology, Biotechnology, Industrial microbiology

## Abstract

The high production costs and unappealing sensory properties still limit the widespread commercialization of microalgae feedstocks. Therefore, this work focused on fine-tuning the heterotrophic medium composition to cultivate novel green, yellow, and white *Chlorella vulgaris* mutant strains. Screening assays were carried out to select the most significant factors, and different nutrient concentrations were optimized by modelling biomass and protein productivity, specific growth rate, and color, via response surface methodology. The biomass and protein productivities achieved by these strains were improved by up to 70% and 94%, respectively. Additionally, biomass color was correlated with medium composition for the first time, allowing the improvement of the yellow and white mutant colorations by 20%. Overall, the findings of this study are vital to overcoming the challenges of the biobased industry, allowing the enhancement of the cost-effectiveness, attractiveness, and nutritional profiles of microalgae-based products in different markets and applications.

**Supplementary Information:**

The online version contains supplementary material available at 10.1038/s41598-025-05913-1.

## Introduction

 According to the United Nations, the world’s population is expected to reach 9.7 billion by 2050^1^, compromising food supply and security worldwide. In this context, traditional livestock farming and agriculture bring forward several environmental concerns, namely the emission of greenhouse gases, land clearing, and habitat degradation^2,3^.

Microalgae are considered an interesting alternative feedstock, owing to their rich nutritional composition and reduced environmental impact^4,5^. The diversity of microalgae is expressed in the variety of compounds they produce, including essential lipids, such as omega-3 fatty acids, high-quality vegan protein, including all the essential amino acids and other important compounds, such as vitamins, namely vitamin B12, iron, zinc and antioxidant pigments^6–8^. The inclusion of microalgal biomass was shown to improve the bioaccessibility and digestibility of some nutrients and/or the composition of food products^8,9^. Furthermore, microalgae can grow on waste streams, requiring less land and water use compared to animal and plant sources, while displaying higher biomass productivity and protein yield than most traditional crops, especially when cultivated under heterotrophic conditions^5,10,11^.

Nevertheless, the industrial production and widespread commercialization of microalgae are challenged not only by their high-costs of production and processing, but also by unappealing sensory properties, like the green color, “grassy” flavor, and “fishy” smell, caused by the presence of chlorophyll^12–14^. Lastly, only a limited number of microalgae species have been approved as novel food, namely *Chlorella* spp. (EU, 2017/2470; EU, 2015/2283). Overcoming these challenges is of vital importance for the competitiveness of microalgal products in the food and feed markets, which relies on designing an appropriate bioprocess, starting with strain improvement and process optimization. Color is of particular relevance in marketing, as it influences consumer´s perceptions towards a product or service, including food^14^. Therefore, its quantification for optimization purposes is of paramount significance.

Different approaches have been used to improve microalgal strains, such as genetic engineering, adaptive laboratory evolution, and random mutagenesis^15^. Random mutagenesis consists of generating random mutations by applying a mutagen to a microalgal culture without introducing foreign genetic material, which exempts these strains from commercialization restrictions related to the generation of genetically modified organisms^15^. After generating a library of mutants, the strains might be screened for target properties and selected by visual evaluation, high-throughput methods, tolerance to environmental stress or exposure to metabolic inhibitors^15–17^.

Regarding process optimization, there is a plethora of abiotic factors and nutrients that considerably affect biomass and target biocompound´s productivity. Abiotic factors like pH and temperature influence several processes of microalgal metabolism^18,19^. Additionally, the sources and concentrations of nutrients such as N, P, Na, K, Mg, Ca, trace elements (e.g., Cu, B, Zn, Mn, Mo, Ni, and Fe) and vitamins can also impact the growth and biochemical composition of microalgae^19–21^. The one-variable at a time (OVAT) approach to optimize a process involves changing one of the variables under study while all others are kept constant and then assessing how the response is influenced by the changed variable, which is highly time- and resource-consuming compared to other approaches^22^. Furthermore, interactions between variables are difficult to estimate systematically^22^. Alternatively, Design of Experiments (DoE) overcomes these limitations by creating models for the optimization of each response^23^. In this case, the workflow usually starts by screening for the most significant factors with fractional factorial experimental designs followed by the determination of the optimal values for a subset of factors with response surface designs, which enables the estimation of interactions and quadratic effects^23,24^.

Considering that the success of microalgae-based products for food and feed applications relies on their nutritional quality, organoleptic traits, and economic viability of the production and processing pipeline, this work focused on the optimization of the abiotic factors (pH and temperature) and medium composition, using a DoE methodology, for a green (8G), a yellow (7Y) and a white (31 W) mutant of *C*. *vulgaris*, all generated by random mutagenesis. In this context, the main goals of the optimization endeavor were to: (i) maximize biomass productivity and growth rate; (ii) maximize protein productivity; (iii) improve the color of the biomass. To the authors’ knowledge, this is the first report of biomass optimization and protein productivity related to the color of heterotrophic microalgae.

## Results and discussion

### Preliminary assays

*Chlorella vulgaris* mutants 8G, 7Y, and 31 W were tested at different temperatures, pH, and with different N sources to establish the optimal parameters towards the conciliation of maximal biomass productivity (*r*_*p*_), growth rate (µ), and protein content (PC) (Table 1).

The responses analyzed concerning the temperature variation were not unanimous among the three mutants. Regarding biomass productivity, mutant 8G did not display differences among the tested temperatures, while the highest productivity in mutant 7Y was achieved at 28 °C (2.70 ± 0.03 g L^−1^ d^−1^) and for mutant 31 W at 30 °C (2.22 ± 0.07 g L^−1^d^−1^). Concerning growth rate, the highest values were achieved at 28 and 30 °C in mutant 8G, at 26 and 28 °C for mutant 7Y, and 30 °C for mutant 31 W. In regard to protein content, mutant 8G displayed the highest value at 28 °C (39.3 ± 0.4% of DW), mutant 7Y at 28 °C and 30 °C (33.6–34.6% of DW), and mutant 31 W at 26 and 30 °C (39.8–41.2% of DW). Since there was no clear optimal temperature for all the responses simultaneously, the temperature of 30 °C was selected, which also corresponds to the temperature at which the mutants were generated.

Concerning pH, mutant 8G did not present differences in biomass productivity and growth rate between pH 5.5 to 7.5. For mutant 7Y, both parameters were higher at pH values of 5.5 and 6.5 (0.75–0.85 g L^−1^d^−1^; 0.80–0.83 d^−1^, respectively), and there were no differences in biomass productivity for mutant 31 W between pH 5.5 and 6.5, while the highest growth rate was attained at pH 6.5 (1.10 d^−1^). In addition, the protein content was higher at pH values of 5.5 and 6.5 for mutant 8G and 7Y, while for mutant 31 W it was higher at 5.5 and 7.5. Given this, a pH value of 6.5 was selected as the optimal condition for the three mutants.

**Table 1:** Biomass productivity (*r*_*p*_) in g L^−1^ d^−1^, growth rate (µ) in d^−1^, and protein content (PC) in % of DW of the three mutants of *C. vulgaris*, 8G, 7Y, and 31 W, tested at 3 temperatures, 3 pH, and with 3 N sources (A – ammonium; N – nitrate; U – urea). Different letters represent statistical differences between the three conditions tested (T, pH, and N source) for each response (*r*_*p*_, µ, PC) for each mutant (*p* < 0.05), i.e., if a response has the same letter under different conditions (of T or pH or N source), there were no significant differences in that response (*r*_*p*_, µ, PC) for that mutant (*p* > 0.05). Results are shown as mean ± s.d., *n* = 3.

		T (°C)			pH			*N* source		
		26	28	30	5.5	6.5	7.5	A	*N*	U
**8G**	***r***_***p***_	1.85 ± 0.02^a^	1.74 ± 0.01^a^	1.85 ± 0.11^a^	2.20 ± 0.07^a^	2.36 ± 0.05^a^	2.18 ± 0.05^a^	2.36 ± 0.05^a^	2.09 ± 0.07^b^	2.29 ± 0.01^a^
	**µ**	1.25 ± 0.01^b^	1.30 ± 0.02^a^	1.31 ± 0.01^a^	1.15 ± 0.01^b^	1.17 ± 0.01^ab^	1.14 ± 0.01^bc^	1.17 ± 0.01^a^	1.13 ± 0.01^b^	1.13 ± 0.01^b^
	**PC**	35.2 ± 0.9^b^	39.3 ± 0.4^a^	37.0 ± 0.9^b^	30.4 ± 0.2^a^	29.3 ± 1.1^ab^	27.1 ± 0.9^b^	29.3 ± 1.1^a^	32.5 ± 1.4^a^	29.2 ± 1.0^a^
**7Y**	***r***_***p***_	2.50 ± 0.03^b^	2.70 ± 0.03^a^	1.93 ± 0.09^c^	0.85 ± 0.06^a^	0.75 ± 0.03^a^	0.42 ± 0.11^b^	0.75 ± 0.03^a^	0.37 ± 0.04^c^	0.64 ± 0.03^b^
	**µ**	1.30 ± 0.00^a^	1.32 ± 0.02^a^	1.05 ± 0.01^b^	0.83 ± 0.02^a^	0.80 ± 0.02^a^	0.66 ± 0.06^b^	0.80 ± 0.02^a^	0.65 ± 0.03^b^	0.77 ± 0.03^a^
	**PC**	32.2 ± 1.1^b^	33.6 ± 0.5^ab^	34.6 ± 0.4^a^	34.9 ± 0.6^a^	35.1 ± 0.1^a^	31.0 ± 1.6^b^	35.1 ± 0.1^a^	27.8 ± 3.9^a^	32.3 ± 2.1^a^
**31 W**	***r***_***p***_	1.71 ± 0.15^b^	1.65 ± 0.06^b^	2.22 ± 0.07^a^	2.06 ± 0.03^ab^	2.48 ± 0.23^a^	1.99 ± 0.05^b^	2.48 ± 0.23^a^	1.48 ± 0.12^b^	2.27 ± 0.06^a^
	**µ**	1.02 ± 0.02^b^	1.01 ± 0.01^b^	1.09 ± 0.01^a^	1.03 ± 0.02^b^	1.10 ± 0.01^a^	1.01 ± 0.03^b^	1.10 ± 0.03^a^	0.88 ± 0.02^b^	1.04 ± 0.03^a^
	**PC**	39.8 ± 1.0^a^	41.2 ± 0.5^a^	33.8 ± 0.5^b^	33.9 ± 0.1^a^	32.1 ± 0.7^b^	35.1 ± 0.3^a^	32.1 ± 0.7^b^	36.3 ± 0.6^a^	36.2 ± 0.3^a^

Finally, the N source that ensured the highest biomass productivity and growth rate was ammonium for the three mutants, although some values did not present considerable differences when compared to urea. In regard to protein content, the three N sources tested enabled to attain similar protein contents, 29.2–32.5% and 27.8–35.1% of DW, in mutants 8G and 7Y, respectively, while mutant 31 W achieved the highest values when cultivated with nitrate and urea, 36.2–36.3% of DW. Since ammonium enabled an improved growth performance among the three mutants, it was selected as one of the N sources to maintain for the three strains. Urea was also included in the screening trials of 8G and 7Y, and nitrate was included in the trials of 31 W instead.

Temperature and pH are critical factors that affect microalgal metabolism, enzymatic reaction rates, and the solubility of nutrients and gases (particularly oxygen), which greatly impact algae growth^25^. The optimal temperature and pH heavily depend on the species used and the goal of the cultivation, as it is possible to deduce by analyzing examples from literature (Supplementary Table S1). *Chlorella vulgaris* is commonly cultivated at 28–30 °C under heterotrophic conditions^25,26^a similar range to the optimal temperatures obtained in the present study. In this work, 30 °C was the temperature selected, not only based on the results obtained for each response, but also because lower cultivation temperatures require more energy to cool down the fermentation broth, which greatly impacts production costs, particularly at larger scales^27^. Regarding pH, the optimal range for microalgal heterotrophic growth is generally between pH 6 and 7^28^. Concerning the impact of these factors on protein content of microalgal cells, information in literature is scarce. Nonetheless, Xie et al.^29^ reported higher protein contents (48–53% of DW) at 28 and 32 °C and at pH 2–5.5 for *Euglena gracilis*.

Nitrogen is also a major macronutrient, since it contributes to the synthesis of amino acids and subsequently proteins. Although microalgae can metabolize different nitrogen sources, ammonium is often preferred, as evidenced in this study, since its uptake requires less energy and for the fact that it can be readily used by the cells without additional reactions, while nitrate and urea need to be converted into ammonium first^19^. Moreover, it has been suggested that ammonium provides a higher proportion of total essential amino acids to total amino acids, including in *C. vulgaris*^29^.

### Screening assays

**Fig. 1:**
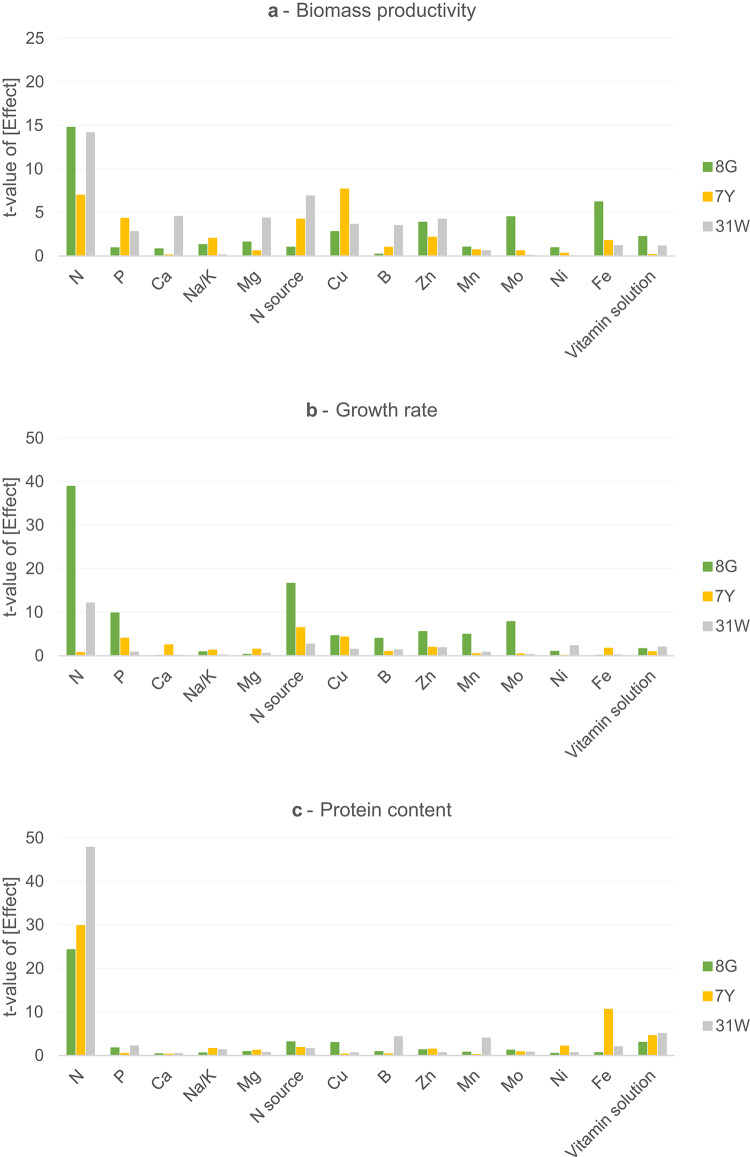
Statistical results obtained in Design Expert v.11.1.2.0: *t*-value of each effect, retrieved from Pareto Charts, for the variables tested in the macro- and micronutrient screening assays (two-level fractional factorial design) for the green (8G), yellow (7Y) and white (31 W) *C. vulgaris* mutants.

Screening assays allow for a reduction of the number of factors in the subsequent optimization trials by removing variables that, in this case, have not had a significant impact on the biomass productivity, growth rate, and protein content. In Fig. 1, a visual representation of the results is shown (a summary of the complete screening trials analysis, namely which factors significantly impacted each response variable (*p* < 0.05) is also available: Supplementary Table S2 and S3).

Overall, the factors that affected growth significantly were the concentration of N, P, Zn, Cu, B, Mn, Mo, Fe and vitamin solution and N source in the case of mutant 8G, the concentration of N, P, Ca, Cu and N source, and Zn was marginally significant for 7Y growth and the concentration of N, P, Ca, Mg, Cu, Zn, Ni and N source for mutant 31 W (Fig. 1a and b). In addition, the factor that most impacted protein content was N concentration across the three screenings, as well as the N source and the concentration of Cu in the case of 8G, the concentration of Fe and the vitamin solution in the case of 7Y, and the concentration of P, B, Mn and vitamin solution for mutant 31 W, but these with a much less significant effect (Fig. 1c).

The three mutants showed considerably improved growth with ammonium instead of urea or nitrate (*p* < 0.05). However, the N source displayed a significant effect on the protein content of mutant 8G. Despite this, the impact of the N source on growth rate was more significant than the effect on protein content (*p* < 0.0001). Thus, N source was excluded as a factor, and the three mutants were optimized using only ammonium as the N source. Then, 4 numeric factors were selected for each optimization, considering the factors with a more significant effect overall. Likewise, the concentrations of N, P, Zn and Mo were the factors selected for the optimization of mutant 8G; the concentrations of N, P, Cu and Zn for mutant 7Y; and the concentration of N, P, Ca and Mg for mutant 31 W.

Kim et al.^30^ also performed a study on the optimization of heterotrophic cultivation of *Chlorella* sp. HS2 towards improved biomass concentration and productivity. In the screening phase (Plackett-Burman Design), the concentrations of N and P were also identified as two of the three key nutrients to optimize. On the other hand, Ward and Rehmann (2019)^31^ reported an optimization for mixotrophic *C. vulgaris* cultivation, where glucose, nitrate, and magnesium were included in a Box-Behnken design. Thus, in these two reports, N, P, and Mg were also significant factors as in the present study.

### Optimization assays

The three mutants’ cultivation conditions were optimized towards improved biomass productivity, growth rate, protein productivity, and color. The equations of the models obtained for each response as well as all the statistical parameters were also obtained (Supplementary Table S4 and S5).

#### Growth performance

**Fig. 2:**
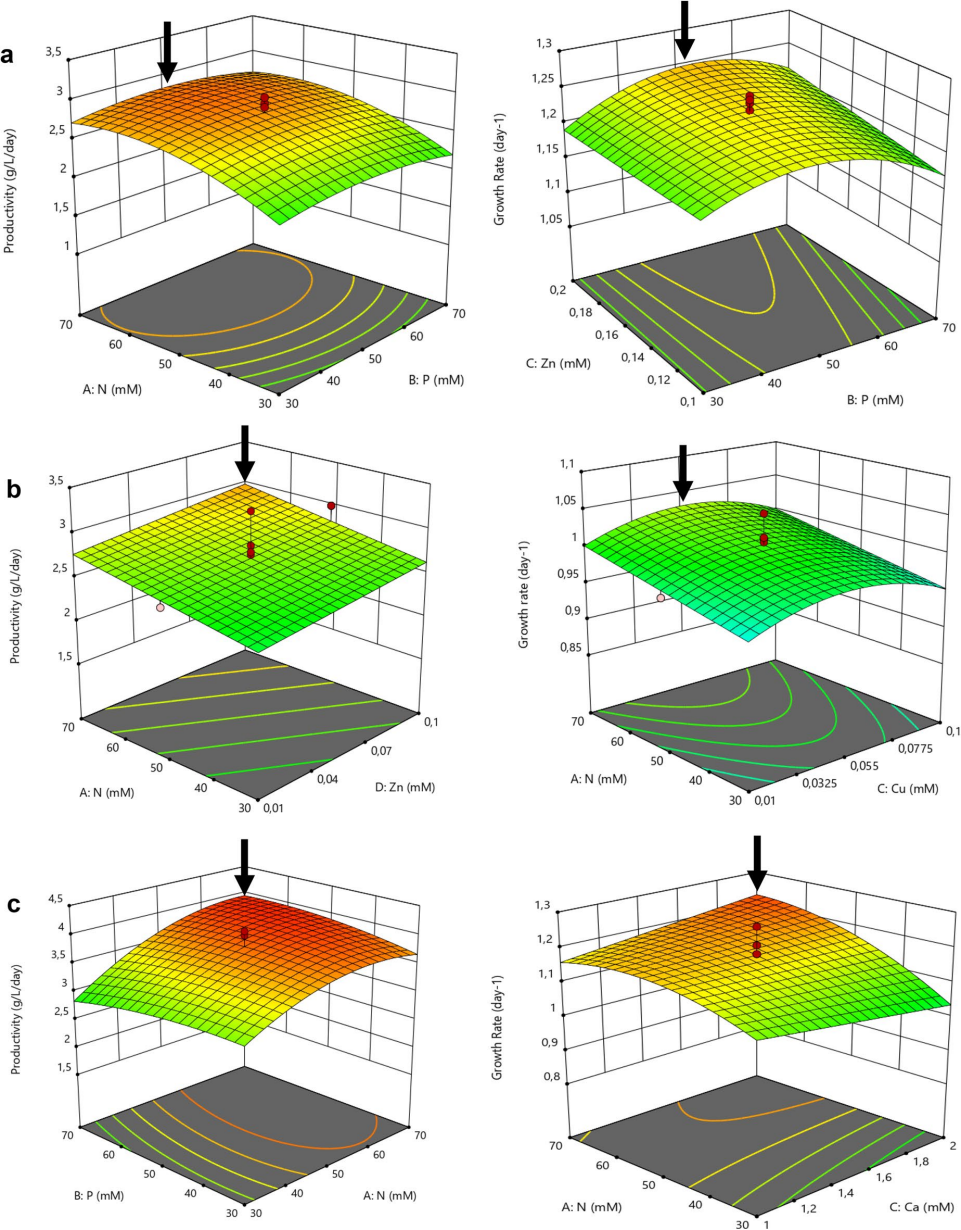
Model Graphics obtained in Design-Expert software (v.11.1.2.0): 3D surface plots of biomass productivity (g L^−1^ d^−1^) and growth rate (d^−1^) predicted for mutant 8G (**a**), 7Y (**b**) and 31 W (**c**) of *C. vulgaris* within the range of the nutrient concentration tested in the optimization trial and the interaction between those 2 factors, considering the average value of the remaining factors tested. The black arrows indicate the conditions in which the highest values of that response would be obtained according to that model. Cooler colors (blue-green) indicate suboptimal conditions, yellow represents intermediate values and warmer colors (orange-red) indicate optimal conditions for that response.

Some of the results obtained for the green, 8G (Fig. 2a), yellow, 7Y (Fig. 2b), and white mutant, 31 W (Fig. 2c) are shown in the figures below, regarding biomass productivity and growth rate.

The biomass productivity model of mutant 8G showed that this parameter was affected by the concentrations of N and Zn and by the quadratic terms of N, P, and Mo, while the growth rate was only significantly affected by the quadratic term of P (*p* < 0.0001). Therefore, improved biomass productivity and growth rate were predicted to achieve the maximum, around 2.7 g L^−1^ d^−1^ and 1.24 d^−1^, respectively, with the highest concentrations of N (60–70 mM) and Zn (0.18–0.20 mM) and intermediate concentrations of P (40–60 mM) and Mo (0.07–0.08 mM) (Fig. 2a). The concentration of N significantly affected both responses for mutant 7Y (*p* = 0.0354 and *p* = 0.055, respectively). Higher concentrations of N (60–70 mM) allowed maximum biomass productivity and growth rate ~ 2.7 g L^−1^ d^−1^ and 1.01 d^−1^, respectively, according to the models obtained (Fig. 2b). Moreover, intermediate concentrations of Cu, between 0.05 and 0.06 mM, also allowed to achieve improved growth parameters (*p* = 0.0003). Finally, Zn also significantly affected biomass productivity (*p* = 0.0425), so that higher values between 0.08 and 0.10 mM led to increased growth.

Mo, Cu, and Zn are beneficial for living organisms at low concentrations but yield negative effects at high concentrations. For example, Mandal et al.^32^ reported enhanced production of reactive oxygen species (ROS) and antioxidant enzymes through the supplementation of Mo (between 0.03 and 0.09 µM). On the other hand, the optimal Cu concentration predicted here (55 µM) was considerably higher than the values reported in literature, ranging from 0.01 to 0.4 µM^30,33,34^. Li et al.^35^ used a Cu concentration of 0.063 mM, close to the optimal concentration determined for mutant 7Y, but achieved a biomass productivity of 0.61 g L^−1^ d^−1^ and a specific growth rate of 0.44 d^−1^, around 5- and 2-fold lower than the predictions of the models generated in this study, respectively, albeit with *Chlorella protothecoides*. On the other hand, Giordano et al.^36^ reported a similar behavior to that was observed here with the variation of Cu concentration, for the cell density of *Chlorella* sp. in heterotrophic conditions. The highest cell concentration was obtained at 0.002 mM of Cu, but at higher concentrations of this metal, the former values rapidly decreased, at least by half. The range of Zn concentrations found in literature, 0.0008 to 0.001 mM, encompasses concentrations lower than the lowest concentration tested in this study, 0.01 mM^30,33,34^. Nevertheless, Shi et al.^33^ cultivated *C. sorokiniana* using a heterotrophic regime with a higher Zn concentration, 0.31 mM, achieving a growth rate similar to the values obtained in the present models, but with a lower biomass productivity.

The biomass productivity of mutant 31 W was impacted by the concentration of N, by the quadratic terms of N and P, and by their interaction (Fig. 2c). In addition, the N concentration, the respective quadratic term, and the interaction of Ca with N and P, significantly affected the growth rate (Fig. 2c). Since it was more beneficial to have Ca between 1.6 and 2.0 mM, according to the interaction with N, then P concentration should be between 60 and 70 mM, according to the interaction with Ca. In resemblance to the green and yellow mutant, N concentrations between 60 and 70 mM provided the highest biomass productivity (3.9 g L^−1^ d^−1^) and growth rate (1.25 d^−1^).

In the present work, increasing concentrations of N resulted in higher biomass productivities, when comparing the low (20 mM) and high (70 mM) N levels. The optimal N concentration predicted here falls within the range of concentrations found in the literature to cultivate *Chlorella* sp. heterotrophically with values ranging from 10 to 94 mM^30,33,34^. Similar results were obtained by Xie et al.^37^. Although these authors used a different N source (sodium nitrate) to cultivate *C. vulgaris*, they also achieved higher DW with higher N concentrations, between 3 and 24 mM. However, under optimal N concentration, the highest DW they achieved was less than 3.5 g L^−1^ over 9 days of cultivation, thus presenting a biomass productivity around 10-fold lower than the maximum reported here. Shen et al.^38^ reached higher values by cultivating *C. protothecoides*, also in heterotrophic conditions, with 60 mM of N (potassium nitrate), attaining 13 g L^−1^ in 8 days, a productivity still 2–3 times lower than the maximum obtained here. In the present study, the biomass productivities and growth rates reported are more than twice the values mentioned above, which could be due to differences in the cultivation methodologies, namely the use of different species and strains, temperatures, glucose concentrations, nitrogen sources and nitrogen concentrations.

While N concentration was consistently more beneficial for growth at the maximum, the three mutants did not present a similar behavior among the different P concentrations. For mutant 8G, intermediate concentrations were preferable (Fig. 2a), while for mutant 31 W, the lowest or the highest concentrations were more favorable, considering the interaction with Ca (data not shown). On the other hand, for mutant 7Y, it was not a significant factor for optimal growth.

It is noteworthy that the range of P concentrations found in the literature to cultivate *Chlorella* sp. in heterotrophic conditions, between 0.2 and 9 mM, is much lower than the lowest concentration used in this study (20 mM)^30,33,34,39^. Li et al.^39^ reported that excess P in heterotrophic conditions, decreased the cell density of *C. regularis* by 40% when cells grew on medium containing 8 mM of P compared to 1.5 mM, causing growth inhibition and cell damage. Overall, these results indicated that while P is an important nutrient for microalgal cell growth, it may result in sub-optimal growth performance if supplied at too high or too low concentrations.

#### Protein productivity

**Fig. 3:**
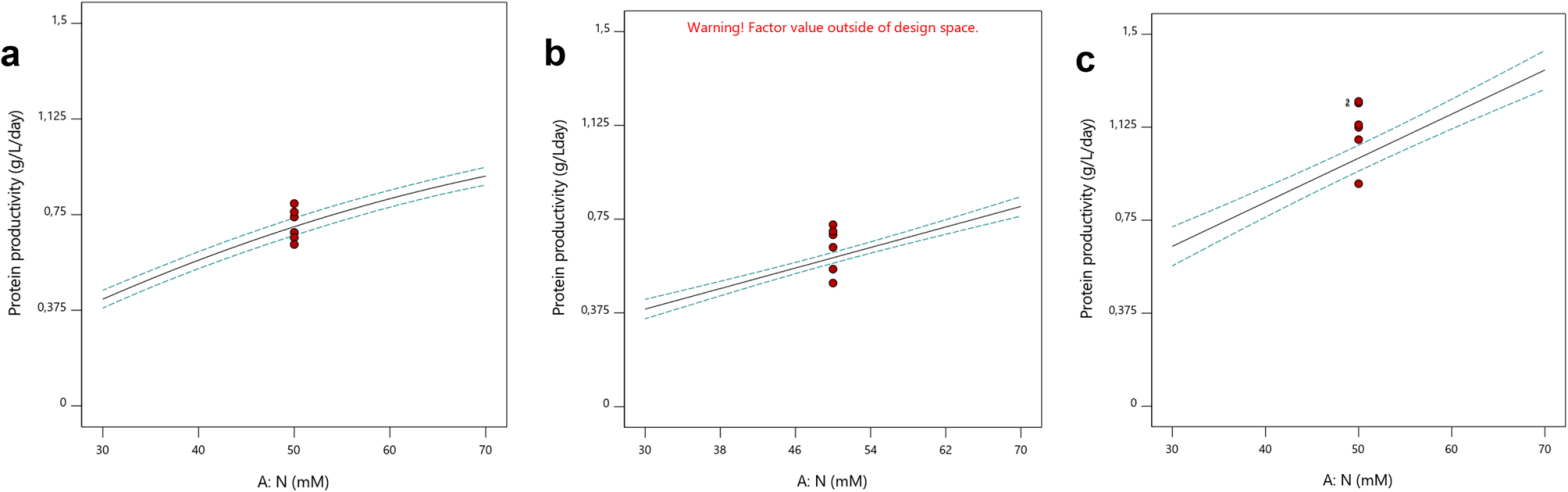
Model Graphics obtained in Design-Expert software (v.11.1.2.0): one-factor plots of protein productivity (g L^−1^ d^−1^) showing the linear effect of changing the concentration of a single factor, N concentration (mM), considering average values of the remaining, for mutant 8G (**a**), 7Y (**b**), and 31 W (**c**) of *C. vulgaris*.

Figure 3 presents the protein productivity results obtained for the green, 8G (a), yellow, 7Y (b), and white 31 W (c) mutants.

Considering the models obtained for each mutant, maximum concentrations of N, around 70 mM, have led to the maximum protein productivities of 0.9, 0.7, and 1.4 g L^−1^ d^−1^, with mutants 8G, 7Y, and 31 W, respectively (Fig. 3). These results are in line with the optimal conditions verified for maximum biomass productivities and growth rates, not only because maximum N concentrations were preferable for the four responses, but also because if higher biomass productivities are achieved, higher protein productivities will be attained.

On the other hand, nitrogen is crucial in the synthesis of amino acids and proteins^19^. Xie et al.^37^ also showed an increment in protein content of *C. vulgaris* at higher N concentrations (but with nitrate instead of ammonium). However, this increment was only observed at concentrations between 3 and 15 mM, which have led to protein contents of 25 and 40% of the DW, respectively. According to these authors, with higher N concentrations, the protein content actually began to decrease slightly. However, in the present study, the protein content and productivity always increased with increasing N concentrations (Fig. 3). It is also noteworthy that if N depletion usually leads to lipid and/or starch accumulation^31,40^as reported for *Chlorella*^41^it is not surprising that an over-compensation leads to protein accumulation, as also suggested by Xie et al.^37^. Despite the different results found, N is undoubtedly one of the nutrients that influence the protein content of microalgal cells more.

Protein contents from 20 to 64% of the DW were reported for *Chlorella* cultivated in heterotrophic conditions^42^. In addition, the quality of *Chlorella* protein, concerning, for example, the amino acid composition, is similar to other protein sources such as fish, eggs, and soybeans^43,44^. It is also noteworthy that *Chlorella* protein might have an added value since the content of other important micronutrients, as iron and vitamin B12, are 10–100-fold higher than in meat, fish, and soy^45–47^.

Despite the fact that lower protein contents were obtained here, compared to the maximum of 64% reported, protein productivity should also be considered, since those authors attained a biomass productivity of 1.65 g L^−1^ d^−145^, which would mean a protein productivity of 1.1 g L^−1^ d^−1^, while in the present work, the biomass productivities were more than twice that, which enabled the achievement of a maximum protein productivity of 1.4 g L^−1^ d^−1^, which is 27% higher than what was reported by those authors.

#### Color

Through the field of colorimetry, it is possible to quantify colors by attributing numerical values to different components according to color systems^48^. The CIE L*a*b* system is considered a standard for color measurement and it has been used in various food applications, as Cairone et al.^49^ discussed in their review.

**Fig. 4:**
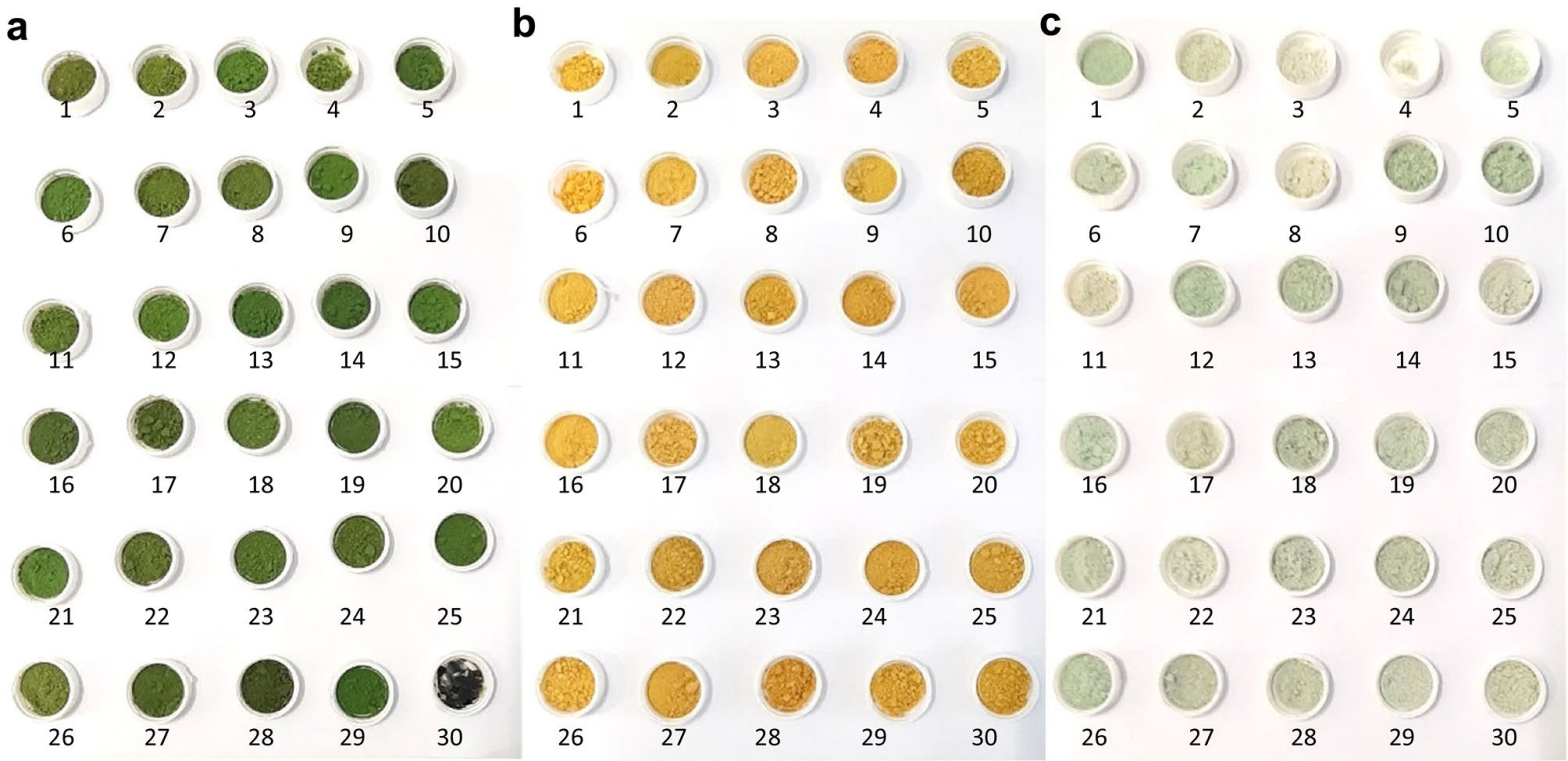
Freeze-dried samples with different coloration of the 30 runs of the optimization trial of the green 8G (**a**), yellow 7Y (**b**) and white 31 W (**c**) mutants of *C. vulgaris*.

**Fig. 5:**
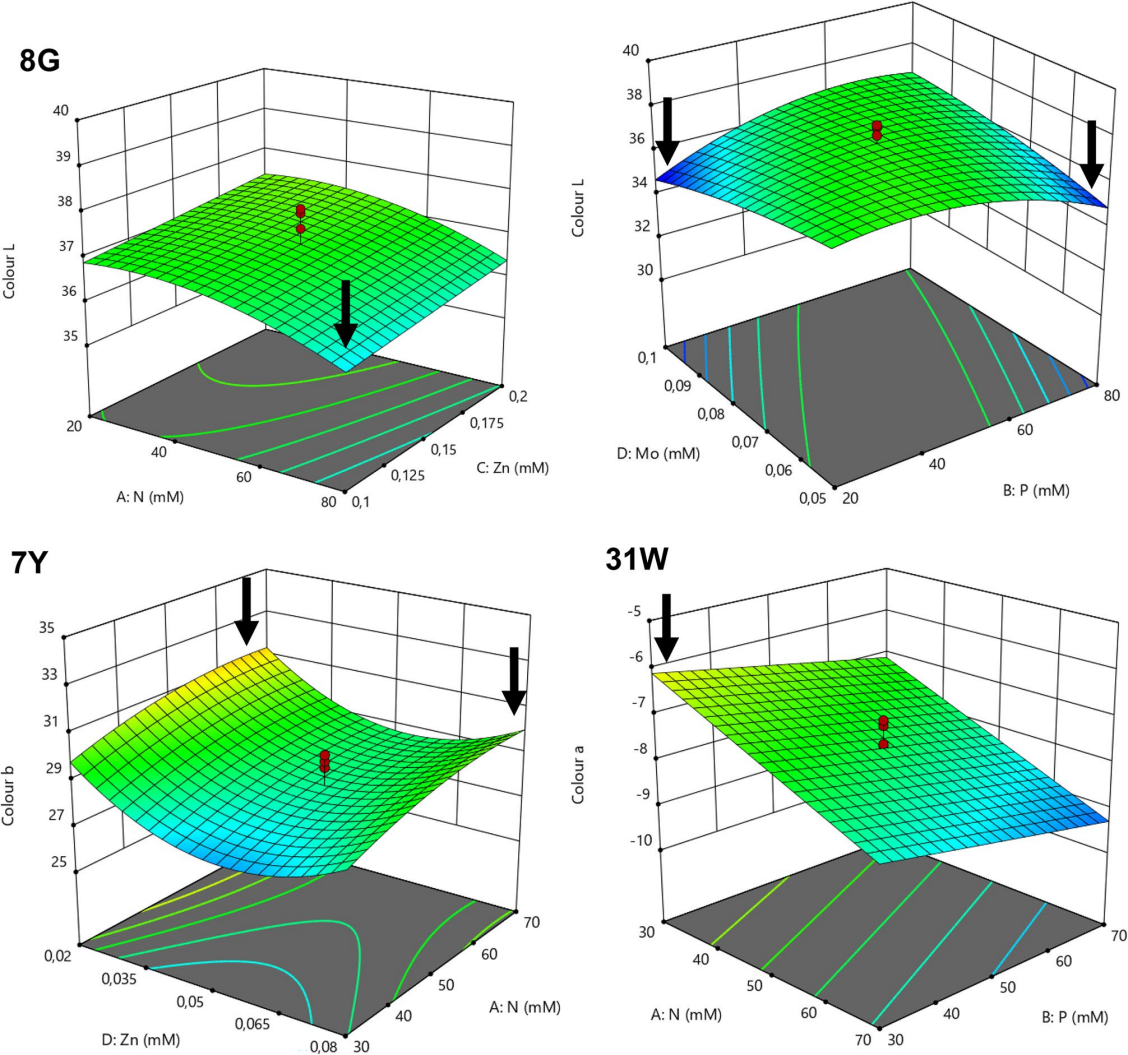
Model Graphics obtained in Design-Expert software (v.11.1.2.0): 3D-surface plots of color: L* (brightness/darkness coordinate) predicted for mutant 8G, b* (yellow/blue coordinate) predicted for mutant 7Y and a* (green/red coordinate) predicted for mutant 31 W of *C. vulgaris* within the range of the nutrient concentration tested in the optimization trial and the interaction between those 2 factors, considering the average value of the remaining factors tested. Cooler colors (blue-green) indicate lower values, yellow represents intermediate values and warmer colors (orange-red) indicate higher values. The darker green color aimed for mutant 8G is represented by the cooler colors, corresponding to lower L* values (darker), a stronger yellow color is represented by warmer colors for mutant 7Y, corresponding to higher b* values (more yellow) and a whiter tone in the case of mutant 31 W is also represented by the warmer colors, corresponding to higher a* values (less green).

Three variables of color characterization were analyzed, L*, a* and b*, but only the most substantial for each mutant was used to create the respective model: L* in the case of mutant 8G (aiming at darker green biomass), b* for mutant 7Y (aiming at stronger yellow colorations), and a* for mutant 31 W (to decrease the greenish tone of the biomass). In Fig. 4, it is possible to observe the color of the samples of the optimization trials (color measurements available: Supplementary Table S6). Color optimization of the green, 8G, yellow, 7Y, and white, 31 W, mutants, is graphically represented in Fig. 5.

Mutant 8G presented a darker green color (lower L*) when higher concentrations of N and lower concentrations of Zn were used. On the other hand, the interactions between N and Mo, P and Zn, P and Mo, and Zn and Mo were all significant (*p* < 0.05). P and Mo had a reversed behavior, when the highest concentrations of Mo were used, the lowest concentrations of P should be applied and vice-versa. Regarding mutant 7Y, N and the quadratic term of Zn affected the yellow color, so that higher concentrations of N and the lowest or the highest Zn concentrations tested were more beneficial for a stronger yellow color (higher b*). Finally, for mutant 31 W the color was predicted to be whiter and/or less green (higher a*) as lower concentrations of N and P were used.

N and P are both macronutrients crucial not only for microalgal growth and metabolism but also for pigment synthesis^20,50^. In addition, the chlorophyll molecule contains four pyrrole groups each with a N atom that form a ring around Mg, responsible for the stabilization of the molecule^50,51^ and ammonium (N source used) is a precursor of glutamate and α-ketoglutarate, which are in turn precursors of chlorophyll biosynthesis^44^. Thus, N content inevitably affects chlorophyll synthesis, so that N deficiency and/or starvation has been reported to cause up to 50% of chlorophyll content reduction in *Chlorella* sp^52,53^. This is in accordance with the models obtained for the color of the green and white mutant, whose green and white color were correlated with high and low concentrations of N, respectively. Similar behavior has been reported for the effect of P in the chlorophyll accumulation of *C. pyrenoidosa*, which might be associated with the requirement of P for ATP synthesis and metabolic processes, such as chlorophyll synthesis^54^. On the other hand, increased Zn concentrations led to decreasing chlorophyll contents, as it has been reported previously for several species, including *C. vulgaris*^55^.

The optimization of color, reported here for the first time for microalgal biomass (to the authors knowledge), is of paramount importance to increase the consumers’ pursuit and acceptance of these products, since it is correlated not only to the visual appearance of the products, but also to the flavor and aroma. This improvement might be the one of the missing pieces to put microalgae in the food market as a top-quality protein source.

### Validation assays

The optimized conditions and the models obtained for each mutant were compared to the standard medium (control condition) to validate the results, and the values attained for each response are shown in Table 2.

**Table 2:** Biomass productivity (*r*_*p*_) in g L^−1^ d^−1^, growth rate (µ) in d^−1^, protein content (PC) in % of DW, protein productivity (PP) in g L^−1^ d^−1^ and color (C) – L* (brightness/darkness coordinate), b* (yellow/blue coordinate) and a* (green/red coordinate) of the three mutants of *C. vulgaris*, 8G, 7Y, and 31 W, respectively and the respective % of change of each solution (S) comparing with the control (Ctl) condition (before optimization). The most interesting solutions have the line highlighted in the table with the color of the respective mutant: green (8G), yellow (7Y) and white (31 W). Different letters indicate statistical differences between the control condition and the solution(s) tested for each response (*r*_*p*_, µ, PC, PP and C) for each mutant (*p* < 0.05). Results are shown as mean ± s.d., *n* = 3. Solutions 1 and 2 tested for mutant 7Y and 31 W included high concentrations of N, while solution 3 was with the lowest N concentration. In solution 2, tested in both mutants, glucose was fed until N depletion, while in solutions 4 and 5, N was gradually added throughout growth.

	S	*r*_*p*_	Change (%)	µ (d^−1^)	Change (%)	PC (%)	Change (%)	PP (%)	Change (%)	C	Change (%)
**8G**	**1**	2.62 ± 0.11^a^	**+ 35**	1.06 ± 0.01^a^	**+ 12**	34.62 ± 0.71^a^	**+ 12**	0.95 ± 0.05^a^	**+ 36**	38.01 ± 0.41^a^	**−2**
	**Ctl**	1.94 ± 0.04^b^	**-**	0.95 ± 0.00^b^	**-**	30.80 ± 0.73^b^	**-**	0.70 ± 0.02^b^	-	38.59 ± 0.26^a^	-
**7Y**	**1**	3.17 ± 0.05^b^	**+ 34**	1.10 ± 0.02^b^	**−4**	40.07 ± 1.12^a^	**+ 57**	1.28 ± 0.06^a^	**+ 94**	32.47 ± 0.25^a, b^	**+ 15**
	**2**	4.03 ± 0.05^a^	**+ 70**	1.16 ± 0.02^a^	**+ 1**	30.27 ± 0.93^b^	**+ 19**	1.21 ± 0.04^a, b^	**+ 83**	33.90 ± 2.02^a^	**+ 20**
	**3**	2.58 ± 0.08^c^	**+ 9**	1.14 ± 0.01^a, b^	**0**	24.00 ± 0.44^c^	**−6**	0.53 ± 0.02^c^	**−20**	28.16 ± 0.47^c^	**0**
	**4**	3.64 ± 0.10^b^	**+ 54**	1.16 ± 0.02^a^	**+ 1**	32.10 ± 1.78^b^	**+ 26**	1.07 ± 0.06^b^	**+ 62**	29.29 ± 1.54^b, c^	**+ 4**
	**Ctl**	2.37 ± 0.08^c^	**-**	1.15 ± 0.01^a, b^	**-**	25.45 ± 0.12^c^	**-**	0.66 ± 0.01^c^	**-**	28.28 ± 0.58^c^	**-**
**31 W**	**1**	2.76 ± 0.12^b^	**+ 30**	1.11 ± 0.00^b^	**+ 1**	37.35 ± 2.28^a^	**+ 60**	1.07 ± 0.07^b^	**+ 41**	−7.23 ± 0.40^a^	**+ 12**
	**2**	3.50 ± 0.05^a^	**+ 65**	1.12 ± 0.01^b^	**+ 2**	30.08 ± 0.10^b^	**+ 29**	0.96 ± 0.01^c^	**+ 26**	−7.64 ± 0.14^a, b^	**+ 18**
	**3**	2.01 ± 0.00^c^	**−5**	1.09 ± 0.00^b^	**−1**	18.05 ± 0.79^d^	**−23**	0.53 ± 0.02^e^	**−30**	−5.20 ± 0.31^d^	**−20**
	**4**	2.86 ± 0.02^b^	**+ 35**	1.11 ± 0.01^b^	**+ 1**	36.53 ± 0.30^a^	**+ 57**	1.06 ± 0.01^b^	**+ 40**	−6.87 ± 0.34^a^	**+ 6**
	**5**	3.36 ± 0.03^a^	**+ 58**	1.16 ± 0.00^a^	**+ 6**	38.24 ± 0.71^a^	**+ 61**	1.29 ± 0.03^a^	**+ 59**	−6.74 ± 0.28^a^	**+ 4**
	**Ctl**	2.12 ± 0.04^c^	**-**	1.10 ± 0.01^b^	**-**	23.33 ± 0.46^c^	**-**	0.76 ± 0.02^d^	**-**	−6.48 ± 0.31^a, c^	**-**

As predicted by the models obtained in the optimization of the three mutants, higher concentrations of N (70, 76 and 67 mM, for mutant 8G, 7Y and 31 W respectively) and the combination of the remaining factors enabled to achieve higher biomass productivities, with improvements of 35% for mutant 8G, 9–70% for mutant 7Y, and 30–65% for mutant 31 W. The highest value was 4.03 ± 0.05 g L^−1^d^−1^, achieved by mutant 7Y. Growth rate followed the same trend but was less pronounced as it is an intrinsic feature of a species. In addition, protein content and productivity also followed this same trend, with maximum improvements of up to 61% and 94%, respectively. The highest protein content was also reached by mutant 7Y, with 40.07 ± 1.12% of DW, and the highest protein productivity was attained by mutant 31 W, with 1.29 ± 0.03 g L^−1^d^−1^. The color of mutant 8G did not present substantial differences between the control and optimized medium condition, while the yellow mutant 7Y presented a 15% and 20% stronger yellow color (b* coordinate) under the conditions of solutions 1 and 2 (with the highest concentration of N – 67 mM), respectively, compared to the WT. Finally, the white mutant 31 W achieved the whitest tone under the conditions of solution 3, with the lowest concentration of N (30 mM), with 20% lower green color (a* coordinate) compared to the WT.

Previous reports highlighted improved growth performances after medium optimization. For example, Kim et al.^30^ optimized the concentrations of nitrate and phosphate and were able to increase the biomass productivity of *Chlorella* sp. by 210% (from 0.84 to 2.59 g L^−1^ d^−1^) compared to the unoptimized medium of that study. The biomass productivities achieved in this study were also higher than the productivity attained by Liang et al.^56^0.15 g L^−1^ d^−1^, that also cultivated *C. vulgaris* heterotrophically. At this scale, Schüler et al.^57^ also cultivated *C. vulgaris*, yielding biomass productivities between 2.45 and 3.23 g L^−1^ d^−1^, while Cheng et al.^58^ were able to attain 3.50 g L^−1^ d^−1^ with *C. protothecoides*. Similarly, Xiong et al.^59^ and Cheng et al.^58^ reported lower specific growth rates (0.44 and 0.58 d^−1^, respectively) than the values obtained in this work. Compared to the present study, O’Grady & Morgan^60^ reported a considerably higher growth rate in *C. protothecoides* (2.30 d^−1^). This suggested that further optimization may be achieved, namely by scaling up the process, as supported by the productivities reported by Barros et al.^61^ of 27.5–31.9 g L^−1^ d^−1^, in 0.2- and 5-m^3^ reactors. Protein content might possibly be further improved also upon scale-up by including, for example, two-stage processes and optimizing feeding strategy as suggested by Xie et al.^37^which reached 44.3% of DW with an N overcompensation two-stage strategy. While the latter was the only study found regarding the optimization of protein with *C. vulgaris*, no study has been found concerning the optimization of the color of the biomass, only of the pigments content, as discussed above.

## Conclusions

The heterotrophic medium of three mutant strains of *C. vulgaris* was optimized by response surface methodology. Although the N concentration was the most significant factor affecting protein content and productivity, as well as biomass color, the concentrations of P, Zn, Mo, Cu, Ca and Mg also significantly impacted the established models. Overall, this work identified the optimum nutrient composition for the precise cultivation of heterotrophic *C. vulgaris*, leading to improved biomass and protein productivities as well as improved color of the final product, which is crucial to ensure the successful commercialization of microalgae-based products in the food market.

## Materials and methods

### Microalgal strains and cultures’ maintenance

Axenic *Chlorella vulgaris* cultures were obtained from Allmicroalgae Natural Products S.A. culture collection. The wildtype strains, 7, 8, and 31, were retrieved from cryopreserved aliquots stored in liquid nitrogen (–196 °C).

The three mutants studied throughout this work were generated by random mutagenesis with ethyl methanesulfonate (EMS), following the protocol reported by Trovão et al.^42^. The green mutant, 8G, was obtained as described by Trovão et al.^62^ by fluorescence-activated cell sorting (FACS). The wildtype strain 7 was mutagenized with EMS (125 mM), which allowed to select the yellow mutant, 7Y, with nicotine (2.5 mM). Finally, the white mutant, 31 W, was attained through 2-round mutagenesis. In the first one, mutant 31W62 was isolated as reported by^42^. This colony was then subjected again to subsequential mutagenesis with EMS (125 mM), which allowed the isolation of mutant 31 W after applying norflurazon (15 µM).

The inoculum of each mutant strain was maintained in PCA plates that were then passed to 250-mL Erlenmeyers, with 50 mL of a proprietary standard heterotrophic medium described by Barros et al.^61^ (HM-medium), which will be referred to later as the control condition for mutant 7Y and 31 W. The same medium was used as control condition for mutant 8G, with slight adjustments in N, P and Zn concentrations: 57 mM, 60 mM and 0.1 mM.

### Growth assessment

Cultures were sampled and analyzed daily by observation through optical microscopy and measurement of the optical density at 600 nm (OD_600_) (Genesys 10 S UV-Vis^®^; Thermo Fisher Scientific, Massachusetts, EUA) and pH (pH 110^®^, VWR, Leicestershire, United Kingdom).

The dry weight (DW) was determined by washing microalgal suspensions with demineralized water and filtering with pre-weighed 0.7 μm glass microfiber filters (VWR International, Pennsylvania, USA). Finally, the samples were dried and weighed at 120 °C using a moisture analyzer (MA 50.R Moisture Analyzer, Radwag^®^, Radom, Poland). The DW (g L^−1^) was calculated by subtracting the weight of the filter ($\:{m}_{i}$, g) from the filter with dried sample ($\:{m}_{f}$, g), and dividing by the volume of filtrated cell suspension ($\:V$, L), according to the following Eq. (1):

1 $$\:DW=\:\left({m}_{f}-{m}_{i}\right)\:/\:V$$.

The *DW* was estimated by establishing a correlation with *OD*_600_ for each *C. vulgaris* mutant: 8G (Eq. (2); *R*^2^ = 0.9757), 7Y (Eq. (3); *R*^2^ = 0.9815) and 31 W (Eq. (4); *R*^2^ = 0.9906):

2 $$\:OD\:\:\left(600\:\text{n}\text{m}\:\right)=2.6258\:\times\:DW$$

3 $$\:OD\:\:\left(600\:\text{n}\text{m}\:\right)=1.8025\:\times\:DW$$

4 $$\:OD\:\:\left(600\:\text{n}\text{m}\:\right)=1.9248\:\times\:DW$$

The biomass productivity (*r*_*p*_, g L^−1^ d^−1^) and the specific cell growth rate (*µ*, d^−1^) of the microalgal cultures were calculated according to the following Eqs. (5) and (6):

5 $${r}_{p}=\left({DW}_{f}-{DW}_{i}\right)\:/\:({t}_{f}-{t}_{i})$$

6 $$\mu\:=\text{ln}({DW}_{f\:}/\:{DW}_{i})/\:({t}_{f}-{t}_{i})$$

where $\:{DW}_{i}$ and $\:{DW}_{f}$ represent the DW of the cultures at the beginning and end of the exponential phase, at the time (d) points $\:{t}_{i}$ and $\:{t}_{f}$, respectively.

At the end of each assay, pellets were frozen at − 20 °C after centrifuging at 4500 *g* for 5 min (Hermle^®^ Z300 centrifuge, Gosheim, Germany). Afterwards, samples were freeze-dried in a Coolvacuum, Lyomicron (Barcelona, Spain), and stored at − 20 °C for biochemical analysis at a later time.

### Laboratory trials

All trials were carried out in triplicate, except in the screening and optimization assays, where a single replicate per experimental condition was used (excluding the central points of the DoE design).

In all the experimental trials, PIPES buffer was added at 75 mM to maintain pH at 6.5. The assays were carried out in 250-mL Erlenmeyers, with 50 mL of final volume, during 5 to 8 days. Cultures were kept in the dark, at 30 °C and 200 rpm, in an orbital shaker (Agitorb 200IC, Norconcessus^®^, Ermesinde, Portugal).

#### Preliminary assays

To determine the optimal temperature, pH, and nitrogen source for each mutant strain, *C*. *vulgaris* 8G, 7Y, and 31 W strains were cultivated in the conditions summarized below (Supplementary Table S7).

Temperature was tested at 26, 28, and 30 °C, at pH 6.5 in HM-medium. The pH of this medium was set at pH 5.5, 6.5, and 7.5 by adding PIPES buffer to the respective conditions, in HM-medium, at 30 °C. Regarding the nitrogen source trial, ammonium sulfate (A), sodium nitrate (N), or urea (U) were tested at a N concentration of 57 mM for strain 8G, whereas 38 mM was used for strains 7Y and 31 W assays, at pH 6.5 and 30 °C.

#### Screening assays

Two screening assays were performed to understand which macro- and micronutrients significantly affected the biomass productivity, growth rate, and protein content (PC) of the three mutants. In both assays, a DoE approach was followed by using a randomized two-level fractional factorial experimental design matrix of resolution IV, set up in Design Expert v11.1.2.0. The set of variables (i.e., factors) in each assay was tested on two levels (Table 3).

These levels corresponded to a low and a high concentration of a specific numeric factor, except for categorical factors, where the two levels corresponded to different categories (i.e., nitrogen sources). For each screening assay, the experimental design consisted of 16 experimental runs plus 4 center points, totaling 20 runs (Supplementary Table S8 and S9).

The micronutrient composition of the standard HM-medium was kept the same in all runs of the macronutrient screening, while the macronutrient concentrations remained constant in the runs of the micronutrient screening. Additionally, the carbon source (glucose) concentration in all media was set at 30 g L^−1^.

**Table 3:** Factors and levels used in the macro- and micronutrient screening assays for each mutant strain of *C. vulgaris* (8G, 7Y, and 31 W).

Factor	Levels							
Strain	8G			7Y		31 W		
		Macronutrient screening						
**[N] (mM)**	20.0		80.0	20.0	80.0	20.0	80.0	
**[P] (mM)**	20.0		80.0	20.0	80.0	20.0	80.0	
**[Ca] (mM)**	0.50		4.00	0.40	2.00	0.40	2.00	
**[Na]/[K]**	0.30		0.90	0.30	0.90	0.30	0.90	
**[Mg] (mM)**	3.00		12.0	3.00	12.0	3.00	12.0	
**Nitrogen source**	Ammonium		Urea	Ammonium	Urea	Ammonium	Nitrate	
		**Micronutrient screening**						
**[Cu] (mM)**	0.0020		0.2000	0.0060	0.0600	0.0060	0.0600	
**[B] (mM)**	0.0500		0.5000	0.0500	0.5000	0.0500	0.5000	
**[Zn] (mM)**	0.0300		0.3000	0.0300	0.3000	0.0300	0.3000	
**[Mn] (mM)**	0.0200		0.2000	0.0200	0.2000	0.0200	0.2000	
**[Mo] (mM)**	0.0005		0.5000	0.0050	0.5000	0.0050	0.5000	
**[Ni] (mM)**	0.0001		0.0010	0.0001	0.0010	0.0001	0.0010	
**[Fe] (mM)**	0.0100		1.0000	0.0500	0.5000	0.0500	0.5000	
**[Vitamin solution]**	0.2500×		2.5000×	0.2500×	2.5000×	0.2500×	2.5000×	

#### Optimization assays

An optimization assay was performed for each strain, according to the most significant 4 factors pointed out in the screening assays, to maximize the *r*_*p*_, µ, and protein productivity (PP) as well as improve the coloration (C) of the biomass of the three mutants. The final aim of the trials was to obtain a darker green hue for mutant 8G, a more intense yellow color for 7Y, and a whiter tone for 31 W).

A response surface methodology was followed by using a central composite inscribed design, in Design Expert v11.1.2.0. For the mutant 8G, the concentrations of N, P, Zn, and Mo were included in the optimization design, concentrations of N, P, Zn and Cu were optimized for mutant 7Y and, for mutant 31 W, the concentrations of N, P, Ca and Mg were selected (Supplementary Table S10). The experimental design consisted of a total of 30 experimental runs, 16 factorial, 8 axial, and 6 center points. The concentrations of the nutrients that were not included in the optimization were set to the same concentration as the HM-medium. Additionally, the glucose concentration of all media was set at 30 g L^−1^.

#### Validation assays

A validation assay was carried out for each strain to compare the optimized medium obtained with the standard HM-medium (control). The optimized medium was formulated in this assay according to the models obtained in the previous optimization assay. The concentrations of the remaining nutrients (not included in the optimization assays) were at the same concentration as in the HM-medium. In the validation of the optimized medium for mutant 8G, only one condition was tested against the control, since the conditions to maximize growth and protein and to reach optimal color were in accordance. The several conditions tested for mutant 7Y provided different yellow tones and different results concerning biomass and protein productivities. To obtain a whiter color of the biomass, the optimized conditions for mutant 31 W were the opposite of the conditions to reach higher productivities of biomass and protein. Thus, several conditions were tested to analyze what would be the most advantageous combination (Supplementary Table S11).

### Biochemical characterization

#### Protein content

The protein content was determined by elemental analysis (Vario EL III^®^; Elementar Analyzer System; GmbH, Hanau, Germany) according to the manufacturer’s instructions. Protein content was estimated by multiplying nitrogen content by the conversion factor of 6.25^63^.

#### Color

The color of the samples was measured with a Chroma Meter (CR-400–410; Konica Minolta; Nieuwegein; Netherlands) and a Color Data Software CM-S100w (SpectraMagic^TM^NX). In this case, the L*a*b* color space, defined by the Comission Internationale de l’Eclairage (CIE) was used (Observer – 2 degrees; Illuminant - C). L* corresponds to the brightness of the sample (S), ranging from 0 to 100; a* to the green-red coordinate and b* to the yellow-blue coordinate, both with numerical values ranging between − 120 and 120. The standard was a white plate, conveyed by the manufacturer, with the C-illuminant having the following values of L*, a* and b*, respectively: 83.8, 0.3185 and 0.3250.

### Statistical analysis

Statistical analyzes were performed using the R statistical package (v. 4.0.5) for the preliminary and validation assays. Experiments were carried out in biological triplicates, and results were expressed as mean ± standard deviation. Results were analyzed by One-way ANOVA, followed by Tukey HSD *post-hoc* multiple comparisons test at a probability level of 0.05.

For the screening and optimization assays, statistical analyzes were performed with the Design-Expert software (v.11.1.2.0), and a significance of 5% was also considered. The biochemical characterization of these samples was carried out in analytical duplicate replicates due to the absence of biological replicates in these trials.

Data was analyzed for each response variable in the screening assays, namely P, µ, PC, and C. The significance of the explanatory variables of the selected factorial model was assessed by ANOVA as well as the selected models for each response of the optimization trials (the same as in the screening trials plus protein productivity). The best-fitting model was selected for each response, whether linear, quadratic, 2-factor interaction, cubic, or a modified version of one of those.

The adequacy of all these models was also evaluated according to the correlation coefficient (*R*^2^), the adjusted and predicted *R*^2 ^and the “Diagnostics” section of Design-Expert software.

## Electronic supplementary material

Below is the link to the electronic supplementary material.

Supplementary Material 1

## Data Availability

The data generated or analysed during this study are included in this published article (and its Supplementary Information files). Other datasets or raw data generated during and/or analysed during the current study are available from the corresponding author on reasonable request.
